# Descriptions of two azooxanthellate *Palythoa* species (Subclass Hexacorallia, Order Zoantharia) from the Ryukyu Archipelago, southern Japan

**DOI:** 10.3897/zookeys.478.8512

**Published:** 2015-01-28

**Authors:** Yuka Irei, Frederic Sinniger, James Davis Reimer

**Affiliations:** 1 Molecular Invertebrate Systematics and Ecology Laboratory, Graduate School of Engineering and Science, University of the Ryukyus, 1 Senbaru, Nishihara, Okinawa 903-0213, Japan; 2 Environmental Impact Assessment Research Group, R&D Center for Submarine Resources, Global Oceanographic Data Center, Japan Agency for Marine Science and Technology, 224-3 Toyohara, Nago, Okinawa 905-2172, Japan; 3 Sesoko Station, Tropical Biosphere Research Center, 3422 Sesoko, Motobu, Okinawa 905-0227, Japan

**Keywords:** Cave-dwelling, cryptic species, ITS-rDNA, Ryukyu Archipelago, zoantharian

## Abstract

Two new species of zoantharians (Hexacorallia, Zoantharia, Sphenopidae), *Palythoa mizigama* **sp. n.** and *Palythoa umbrosa* **sp. n.**, are described from the Ryukyu Archipelago, southern Japan. Unlike almost all other known *Palythoa* spp., both species are azooxanthellate and inhabit low-light environments such as floors or sides of caves, crevasses, or hollows of shallow coral reefs. The two species were initially considered to be the same species from their similar habitat environments and highly similar morphological features. However, phylogenetic analyses of nuclear internal transcribed spacer (ITS) ribosomal DNA, mitochondrial 16S ribosomal DNA, and cytochrome oxidase subunit I (COI) sequences revealed that these two species have a genetically distant relationship within the genus *Palythoa*. Morphological characteristics, including polyp size, tentacle number, external/internal coloration, and types and sizes of cnidae were examined in this study. As a result, only tentacle coloration was found to be useful for the morphological distinction between the two species. *Palythoa mizigama* possesses white tentacles with black horizontal stripes while *Palythoa umbrosa* possesses white tentacles without any stripe patterns. Considering their distant phylogenetic relationship, it can be assumed that their unique yet similar morphological and ecological characteristics developed independently in each species as an example of parallel evolution.

## Introduction

Zoantharia are sessile marine cnidarians within the class Anthozoa, subclass Hexacorallia. This taxon is often referred to as intermediate in form between hard corals (Scleractinia) and sea anemones (Actiniaria), as most species lack a skeleton and yet are colonial. Zoantharians are widely distributed and are particularly common in subtropical and tropical regions, where they are one of the major benthic components of coral reefs. Some species contain unique chemicals such as palytoxin or norzoanthamine (Kuramoto et al. 1997, Moore and Scheuer 1971, Vitor and Vitor 2010). Although they are important organisms both ecologically and as a source of bioactive compounds, there is a lack of knowledge on their taxonomy and diversity, with both many synonyms and undescribed species existing (Burnett et al. 1997, Reimer et al. 2004). Zoantharians are usually distinguishable by the combination of two rows of tentacles arranged around the oral disc, colonial life form (although there are some exceptions), and the incrustation of hard particles in their column taken from the surrounding habitat (excepting species of the family Zoanthidae). Within Zoantharia, there are two suborders, Macrocnemina and Brachycnemina, which are distinguished by the state of their fifth mesentery from the dorsal directive, being complete or incomplete, respectively. Macrocnemic zoantharians have a wide habitat range from intertidal to abyssal depths, and polar to tropical waters (Ryland et al. 2000). Additionally, many macrocnemic zoantharians live in association with other organisms such as sponges (Crocker and Reiswig 1981), hydrozoans (Camillo et al. 2010, Sinniger et al. 2010), antipatharians (Sinniger et al. 2010), or they are epizoic on shells inhabited by hermit crabs (Muirhead et al. 1986, Reimer et al. 2010, Schejter et al. 2011). On the other hand, brachycnemic zoantharians inhabit shallow waters in subtropical to tropical waters and most species have symbiotic relationships with *Symbiodinium* spp. (e.g. Trench 1974, Burnett 2002, Reimer et al. 2006b). Brachycnemic zoantharians are abundant on many coral reefs (Sebens 1982, Acosta 2001, Irei et al. 2011) and usually have competitive relationships with other benthic organisms (Suchanek and Green 1981).

The suborder Brachycnemina currently contains three families; Sphenopidae, Zoanthidae, and Neozoanthidae. In the Ryukyu Archipelago in southwestern Japan, one genus of family Sphenopidae (*Palythoa* Lamouroux, 1816), two genera of family Zoanthidae (*Isaurus* Gray, 1828, *Zoanthus* Lamarck, 1801), and one genus in family Neozoanthidae (*Neozoanthus* Herberts, 1972) have been reported (Reimer 2010). Additionally, specimens from the genus *Sphenopus* Steenstrup, 1856 have recently been found in the area (T. Fujii, pers. comm.; J. Reimer, unpubl. obs.). These three families are easily distinguished from each other in the field by external morphological features such as presence or absence of sand incrustation, polyp size, and gross morphology (solitary or colonial) (Reimer 2010).

The two genera in the family Sphenopidae, *Sphenopus* and *Palythoa*, can easily be distinguished from each other by morphological and ecological characteristics. Unusually for a zoantharian, *Sphenopus* is azooxanthellate, has a solitary (=unitary, not forming colonies) life form and inhabits muddy or sandy bottoms without usually attaching to hard substrates (Gray 1867, Soong et al. 1999). The validity of the genus *Sphenopus* has recently been questioned since molecular analyses have shown that this group is phylogenetically included within the genus *Palythoa* (Reimer et al. 2012b). According to the World Register of Marine Species, three species have been described in this genus (Reimer 2014a). On the other hand, species of the genus *Palythoa* are colonial and zooxanthellate, similar to most other brachycnemic zoantharians. Some *Palythoa* species are known to compete for space and overgrow other organisms in coral reefs (Suchanek and Green 1981).

In this study, we describe two new *Palythoa* species discovered from the Ryukyu Archipelago, southern Japan. These two species were initially considered to be the same species as they were found in similar environments and exhibit highly similar morphological features. However, molecular analyses demonstrated considerable differences between these two groups and subtle morphological differences distinguish the two groups. Uniquely for *Palythoa*, both species are azooxanthellate and are found in caves and cracks in coral reefs. These results lead to a reconsideration of the definition of both *Palythoa* and *Sphenopus*, and provide an example of parallel evolution within Zoantharia.

## Methods

### Sampling

**Figure 1.**
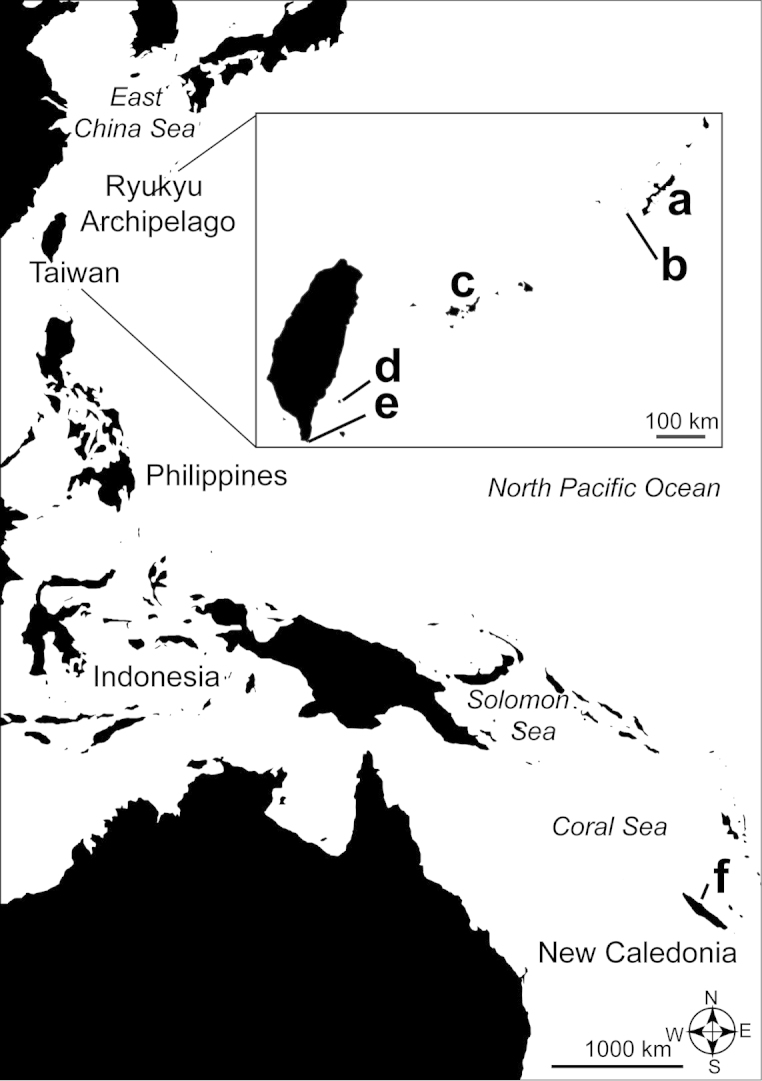
Sampling locations of *Palythoa mizigama* sp. n. and *Palythoa umbrosa* sp. n. specimens. **a** Okinawa Island **b** Tokashiki Island **c** Yaeyama Islands **d** Lyudao (Green Island) **e** Kenting **f** Poindimié. *Palythoa mizigama* was found from Ryukyu Archipelago, Taiwan and New Caledonia (**a, b, c, e, f**), while *Palythoa umbrosa* was found from the Yaeyama Islands and Taiwan (**c, d**).

All specimens were collected between 2008 and 2012 by scuba or snorkeling from depths of 2.6 to 12 m, primarily from the Ryukyu Archipelago, southwestern Japan, with a few additional specimens collected from two locations in Taiwan and one location in New Caledonia (Figure 1, Suppl. material 1: Table 1). Collected specimens were stored in separate vials and fixed in 70–99.5% ethanol for molecular analyses or 4–10% seawater formalin for morphological observations.

Abbreviations of sampling sites:

ADA Ada (Okinawa Island)

IRI Iriomote (Yaeyama Islands)

MIZ Mizugama (Okinawa Island)

NC New Caledonia

ODO Odo (Okinawa Island)

SUN Sunabe (Okinawa Island)

TEN Teniya (Okinawa Island)

TOK Tokashiki (Kerama Islands)

TW Taiwan

YON Yona (Okinawa Island)

### Molecular analyses

DNA extraction was performed with guanidine following the protocol described in Sinniger et al. (2010). Three DNA markers; mitochondrial cytochrome oxidase subunit I (COI), mitochondrial 16S ribosomal gene (mt 16S rDNA), and the nuclear internal transcribed spacer region of ribosomal DNA (ITS rDNA), were amplified by polymerase chain reaction method with HotStarTaq Master Mix Kit (Qiagen, Tokyo, Japan). The COI region was amplified by using the zoantharian-specific primer COzoanF (Reimer et al. 2007a) and universal HCO2198 (Folmer et al. 1994). The primers 16Santla and 16SbmoH (Sinniger et al. 2005) were used for mt 16S rDNA, and either the primer pair of ITSfSW and ITSrSW (Swain 2009) or ZoanF and ZoanR (Reimer et al. 2007b) was used to amplify ITS rDNA. PCR products were purified by using shrimp alkaline phosphate (SAP) and Exonuclease I (Takara Bio Inc., Shiga, Japan) and then sequenced either by Macrogen Japan Corp. (Tokyo, Japan) or Fasmac Co., Ltd. (Kanagawa, Japan). Sequences obtained were automatically aligned along with sequences from GenBank (National Center for Biotechnology Information) by MUSCLE (Edgar 2004) and manually inspected by Se-Al v2.0a11 Carbon (Rambaut and Charleston 1997–2001). Phylogenetic trees were constructed by three methods; maximum likelihood (ML), neighbor joining (NJ), and Bayesian posterior probability (Bayes). ML trees were made by PhyML Online (Guindon et al. 2010) under the GTR (general time-reversible model) substitution model. The number of substitution rate categories was set for eight and bootstraps were calculated with 1000 replicates. NJ trees were constructed by using Clustal X 2.1 (Larkin et al. 2007) with 1000 bootstrap replicates. Bayesian trees were constructed in MrBayes 3.1.2 (Ronquist and Huelsenbeck 2003) under the GTR + I + I^-^ model. One cold and three heated Markov chain Monte Carlo (MCMC) chains with default-chain temperatures were run for two million generations, sampling log-likelihoods (InLs), and trees at 100-generation intervals (20,000 InLs and trees were saved during MCMC). The first 2,000 generations were discarded as “burn-in”, and Bayesian posterior probabilities and branch-lengths were estimated from the remaining 18,000 generations.

### Morphological observations

Color patterns were noted from images taken in situ or observed under stereomicroscope. Relative tentacle length (i.e. ratio of tentacle length to oral disk diameter) was estimated from images taken in situ. Additional in situ images (not shown) were taken with small rulers placed next to colonies and polyps. Height (length) and diameter of polyp column (width) of individual polyps were measured by using calipers after fixation, and numbers of tentacles were counted under stereomicroscope. Additionally, cnidae were observed, characterized and measured from three parts of each polyp; tentacles, pharynx, and mesenterial filaments. Portions of each tissue were removed and put on a separate slide glass and a cover glass was gently placed over tissue after adding a drop of glycerin solution (seawater:glycerin=1:1). The cover glass was slightly pressed to squash and disperse tissue and the edges of the cover glasses were sealed by clear nail polish. Prepared slides were then observed under a differential interference contrast (DIC) microscope (Nikon Eclipse80i, Nikon, Tokyo) and photographs of each type of cnidae were taken with a digital camera (Canon Powershot G11, Canon, Tokyo). Types of cnidae were determined with reference to Ryland and Lancaster (2004) and length and width of each cnida was measured by using ImageJ software (Rasband 1997–2012).

## Results

### Systematics Phylum Cnidaria Hatschek, 1888 Class Anthozoa Ehrenberg, 1831 Subclass Hexacorallia Haeckel, 1896 Order Zoantharia Gray, 1832 Suborder Brachycnemina Haddon & Shackleton, 1891 Family Sphenopidae Hertwig, 1882

#### Genus Palythoa

Taxon classificationAnimaliaZoanthariaSphenopidae

Lamouroux, 1816

##### Type species.

*Palythoa mammillosa* (Ellis & Solander, 1786).

##### Description.

Colonial brachycnemic zoantharians with heavily sand-incrusted ectoderm and mesoglea. Occasionally solitary polyps are also seen.

##### Remarks.

Various authors have suggested to include the genus *Protopalythoa* Verrill, 1900 over the past century based on both morphological (Pax 1910, Ryland and Lancaster 2003) and molecular data (Burnett et al. 1997, Reimer et al. 2006a). Polyps of *Palythoa* are embedded in a well-developed coenenchyme (= “immersae”, Pax 1910) while those of *Protopalythoa* are more free and clear of the coenenchyme (= “intermediate” or “liberae”, Pax 1910). In this study, we consider *Protopalythoa* to be included within *Palythoa*. In general, polyps are firmly attached to stones or reef rocks. Currently, approximately 130 nominal species have been described (*Palythoa* + *Protopalythoa*, Reimer 2014b, c) although some studies have suggested the presence of many synonyms (Burnett et al. 1997, Reimer et al. 2004, Reimer et al. 2012a). All described species are zooxanthellate with the exception of *Palythoa macmurrichi* (Haddon & Shackleton, 1891) (see Remarks below).

#### Palythoa mizigama sp. n.

Taxon classificationAnimaliaZoanthariaSphenopidae

http://zoobank.org/097C1C97-02B2-48FA-8E42-25942ECA982D

Figures 1 , 2 , 3 , 4 , 5 , 7 , 8 , 9 , Suppl. material 1 –2 : Table 1 –2

“Palythoa sp. tokashiki” – Reimer 2010, Reimer et al. 2011b (specimen from Kenting, Taiwan), Reimer et al. 2013 (specimen from Kenting). Synonymy.

##### Type material.

**Type-specimens.** Holotype. Specimen number NSMT-Co1560 (original number MIZ_33). Fixed in 99.5% ethanol, deposited in National Museum of Nature and Science, Tokyo, Japan. Original label: “HOLOTYPE *Palythoa mizigama*, Japan, Okinawa Island, Kadena, Mizugama, 5 m depth, 13 May 2008, Y. Irei leg. Paratypes: Paratype 1. Specimen number RMNH Coel. 41729 (original number TOK_2), Japan, Okinawa Prefecture, Kerama Islands, Tokashiki Island, Mutizuni, 26°09'07"N, 127°21'11"E, in a hollow of reef slope at 5 m depth, 5 January 2008, Y. Irei and F. Sinniger leg. Fixed in 99.5% ethanol, deposited in Naturalis Biodiversity Center, Leiden, The Netherlands. Paratype 2. Specimen number USNM 1231375 (original number ODO_25), Japan, Okinawa Prefecture, Okinawa Island, Itoman, Odo, 26°05'06"N, 127°42'32"E, on the wall of reef cave at 11 m depth, 13 January 2008, Y. Irei and F. Sinniger leg. Fixed in 99.5% ethanol, deposited in Smithsonian Institution National Museum of Natural History, Washington, D.C., USA. Paratype 3. Specimen number RUMF-ZG-04375 (original number IRI_JR2829), Japan, Okinawa Prefecture, Yaeyama Islands, Taketomi, Iriomotesuido, 24°21'51"N, 123°57'25"E, at 4.4–5.3 m depth, 1 September 2012, J. Reimer leg. Fixed in 99.5% ethanol, deposited in University Museum, University of the Ryukyus (Fujukan).

##### Type-locality.

Japan, Okinawa Prefecture, Okinawa Island, Kadena, Mizugama, 26°21'35"N, 127°44'18"E, on wall of reef cave at 5 m depth, 13 May 2008, Y. Irei leg.

##### Description of holotype.

Size of colony approximately 2 cm × 3 cm, consisting of six polyps, 3.8–11.2 mm in height and 2.0–3.8 mm in diameter. External color brownish gray to yellowish white with small black blotches on polyp heads and columns. Horizontal wrinkles (1–7 in number) of approximately half the length of column periphery, mostly on inner side of bent polyps. Tentacles approximately 32 in number, color same as column with 7–10 narrow black horizontal lines on tentacles. Columns heavily incrusted with irregularly-sized sand grains.

##### Habitat and ecological features.

**Figure 2.**
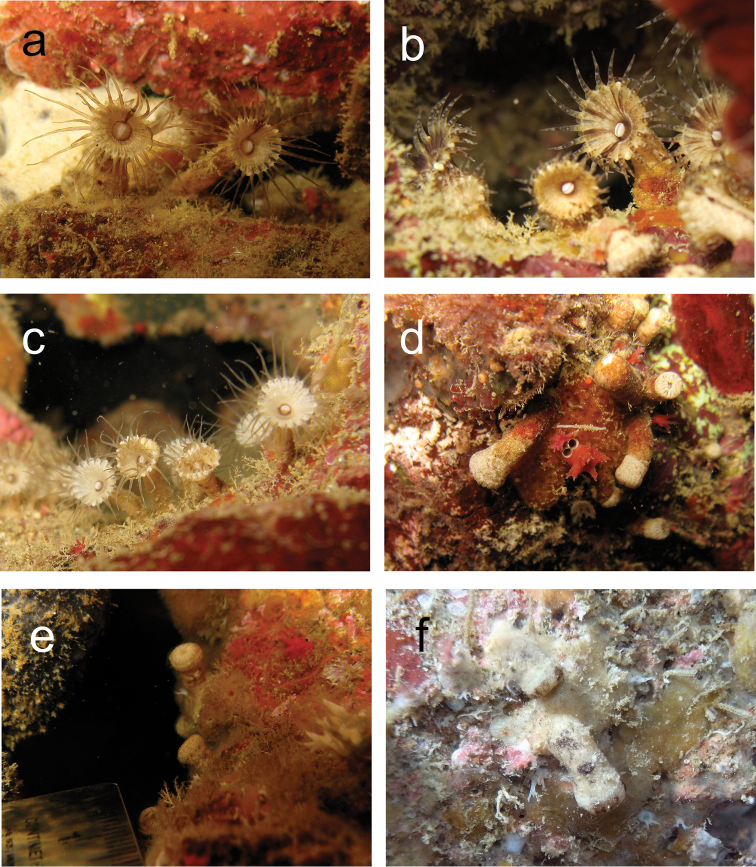
In situ images of *Palythoa mizigama* sp. n. and *Palythoa umbrosa* sp. n. **a** *Palythoa mizigama*; single radial lines visible on oral disks. Image taken by Y. Irei on September 28, 2009, at Mizugama, Okinawa Island, Japan **b** *Palythoa mizigama*; several radial lines visible on oral disks. Image taken by J. Reimer on April 22, 2010, at Mizugama **c** *Palythoa mizigama*; with no radial lines on oral disks. Image taken by Y. Irei on April 12, 2009, at Mizugama **d** *Palythoa mizigama*; closed polyps. Image taken by Y. Irei on June 16, 2009, at Mizugama **e** *Palythoa umbrosa* (TW_18); closed polyps. Image taken by Y. Irei on November 2, 2009, at Lyudao, Taiwan **f** *Palythoa umbrosa* (IRI_TF2); closed polyps. Image taken by T. Fujii on November 9, 2012, at Sotobanari Island, Yaeyama Islands, Japan.

This species inhabits low-light environments such as floors or sides of caves, crevasses or under reef overhangs on coral reef flats and reef slopes. In general, polyps are open at night with extended tentacles, and are closed in daytime. Polyps tend to bend parallel to the surrounding substrate when closed although polyps become somewhat erect in a diagonal direction at an acute angle (e.g. not perpendicular to substrate) when open, with oral disks generally facing the opening of the cave or crevasse (Figure 2a–d).

##### Distribution.

Southern Ryukyu Archipelago (Okinawa Island, Tokashiki Island, Yaeyama Islands), Taiwan (Kenting), and New Caledonia (Tibarama, Poindimié) (Figure 1).

##### Diagnosis.

**Figure 3.**
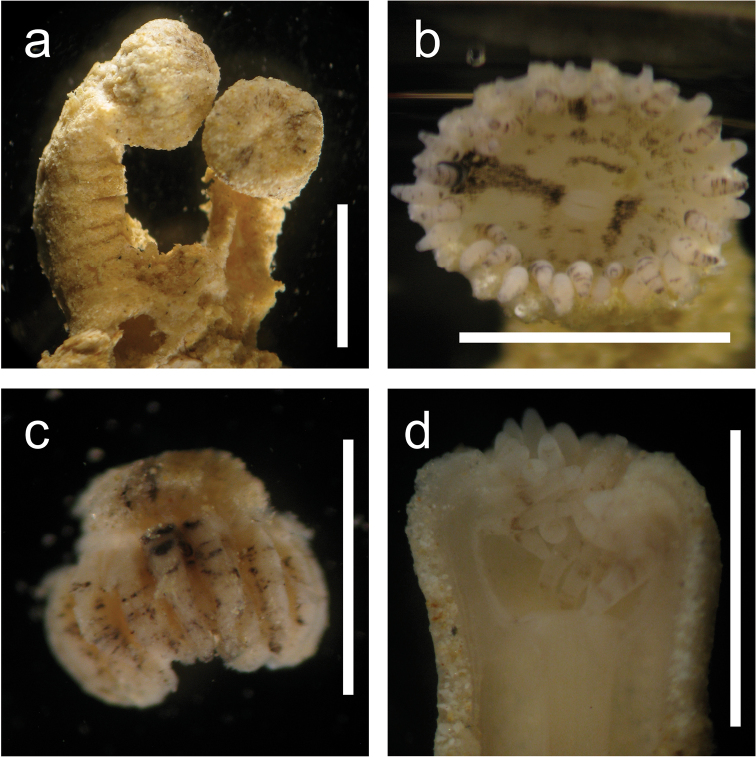
*Palythoa mizigama* sp. n. after fixation in 70–99.5% ethanol (**a, c**) or 4–10% SW formalin (**b, d**). **a** MIZ_33; closed polyps. Scale bar=0.5 cm. Collected by Y. Irei on May 13, 2008 at Mizugama, Okinawa Island, Japan **b** MIZ_071110_2; close up of a partially open polyp. Scale bar=0.5 cm. Collected by Y. Irei on November 7, 2010 at Mizugama **c** MIZ_33; tentacles (isolated). Horizontal black stripes visible. Scale bar=0.25 cm **d** MIZ_260910; close up image of a longitudinal section. Tentacles visible with faint stripes. Note the column wall densely incrusted with sand grains. Scale bar=0.25 cm. Collected by Y. Irei on September 26, 2010 at Mizugama. All images taken through stereomicroscope.

*General.* Azooxanthellate brachycnemic zoantharian with heavily sand-incrusted ectoderm and mesoglea. Colonies usually composed of several to 20 polyps, with each polyp loosely connected to others by a thin stolon (=“liberae”, Pax 1910). Solitary polyps are also commonly seen. Polyp is cylindrical and upper part of the polyp around pharynx is occasionally constricted when closed (Figure 3a). Expanded polyps are flared, with column becoming wider towards the oral disk and a large oral disk (Figure 2a, c). Columns occasionally have several horizontal wrinkles (1 to up to 10 in number) of one quarter to half the length of column periphery, mostly on inner side of bent polyps (Figure 3a).

*Polyp size.* Approximately 0.5–1.0 cm in length and 0.2–0.4 cm in width after fixation in 4–10% seawater formalin or 70–99.5% ethanol.

*Coloration of polyp column and oral disk.* The color of external polyp columns varies from ivory to tan in life, occasionally with irregular black blotches. The color of oral disk is also ivory to tan, often with black radial lines extending outwards from the mouth (Figure 2 a and b, Figure 3 b).

*Tentacles.* 32–40 in number. Extended tentacles are of the same length or longer than the diameter of oral disks (Figure 2a, c). Tentacle color is white to ivory with 4–10 horizontal black stripes (Figure 3b). The number and density of stripes on the tentacles are variable between different polyps (Figure 3c, d).

**Figure 4.**
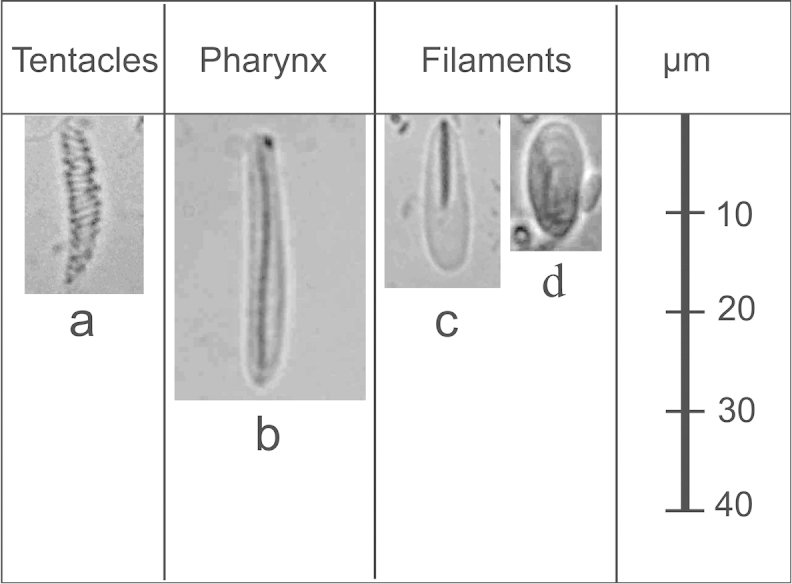
Dominant cnidae types of *Palythoa mizigama* sp. n. and *Palythoa umbrosa* sp. n. tissue. **a** spirocyst **b** basitrich, and **c** *p*-mastigophore **d** holotrich. Scale bar shows 10–40 μm.

*Cnidom.* Four major cnidae types observed; spirocysts, basitrichs, *p*-mastigophores, and holotrichs. The dominant types of cnidae were spirocysts (length 20.5±3.5 μm, width 3.4±0.7 μm, n=140) in the tentacles, basitrichs (length 23.7±2.3 μm, width 3.6±0.4 μm, n=139) in the pharynx, and *p*-mastigophores (length 15.7±3.6 μm, width 4.7±0.7 μm, n=140) and holotrichs (length 13.5±1.9 μm, width 4.9±0.7 μm, n=140) in the mesenterial filaments (Figure 4, Suppl. material 2: Table 2). In addition to the four major cnidae types, three large holotrichs were found. Three individual polyps contained one large holotrich each, with two from tentacles (length 28.4 μm, width 13.0 μm; length 25.7 μm, width 11.7 μm) and one from the mesenterial filaments (length 33.4 μm, width 16.9 μm).

##### Etymology.

The specific epithet “mizigama” was named after the type locality of Mizugama. Additionally, “mizi” and “gama” mean “water” and “cave”, respectively, in the Okinawan language. Japanese name: Shimate-yami-iwasunaginchaku. “Shimate”, “yami”, and “iwasunaginchaku” mean “striped hands”, “dark”, and “*Palythoa*”, respectively, in Japanese.

##### Remarks on reproduction.

**Figure 5.**
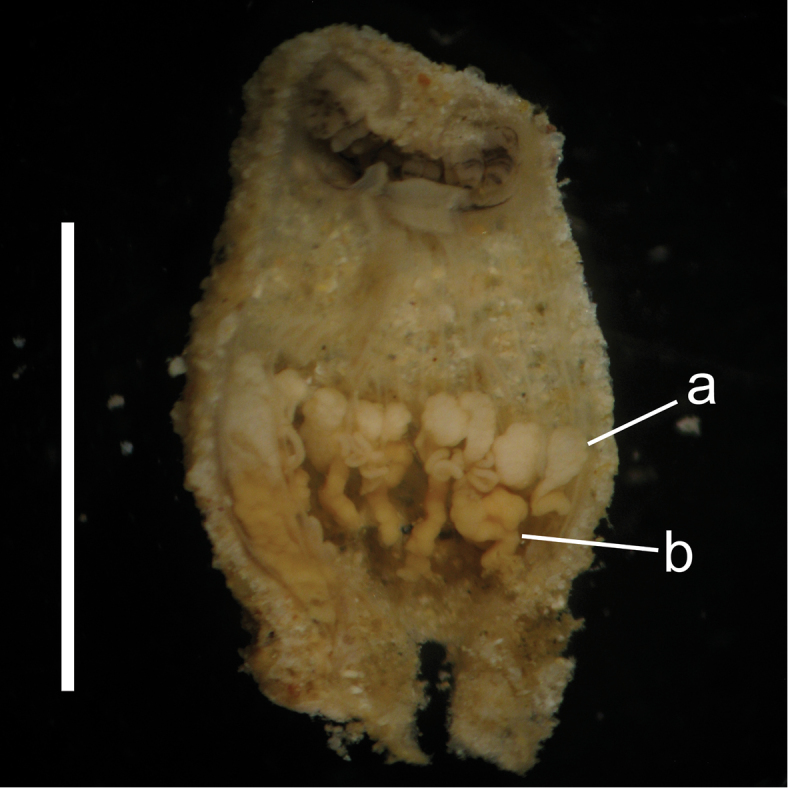
Longitudinal section of a reproductive polyp of *Palythoa mizigama* sp. n. Collected on Aug. 19, 2010 at Mizugama, Okinawa Island, Japan. **a** spermaries **b** ovaries. Scale bar=0.5 cm.

Simultaneous hermaphrodite. Reproductive polyps containing ovaries and spermaries were found from specimens collected in August - September 2010 at Mizugama, Okinawa Island, Japan (Figure 5).

#### Palythoa umbrosa sp. n.

Taxon classificationAnimaliaZoanthariaSphenopidae

http://zoobank.org/6DC531A5-26E6-432F-A726-3A6F15B9D8BF

**Figure 6.**
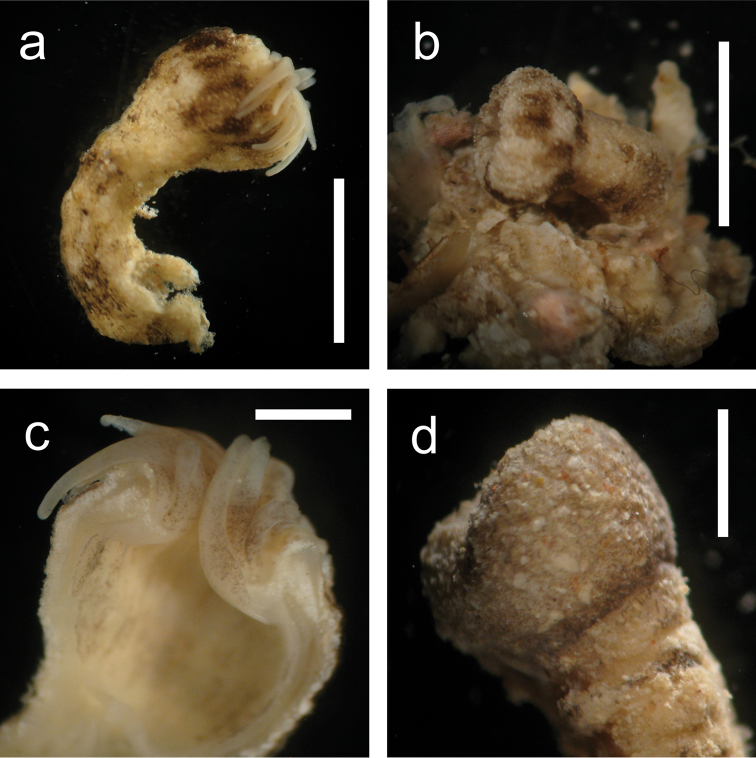
*Palythoa umbrosa* sp. n. after fixation in 4-10% SW formalin. **a** IRI_TF2; slightly expanded polyp. Scale bar=0.5 cm. Collected by T. Fujii on November 9, 2012 at Sotobanari Island, Yaeyama Islands, Japan **b** IRI_TF1; closed polyp. Scale bar=0.5 cm. Collected by T. Fujii on November 9, 2012 at Sotobanari Island **c** IRI_TF2; close up of a longitudinal section. Note the white tentacles with small black dots. Scale bar=0.2 cm **d** IRI_TF2; close up of a polyp capitalum. Column incrusted with irregularly-sized particles. Scale bar=0.25 cm. All images taken through stereomicroscope.

**Figure 7.**
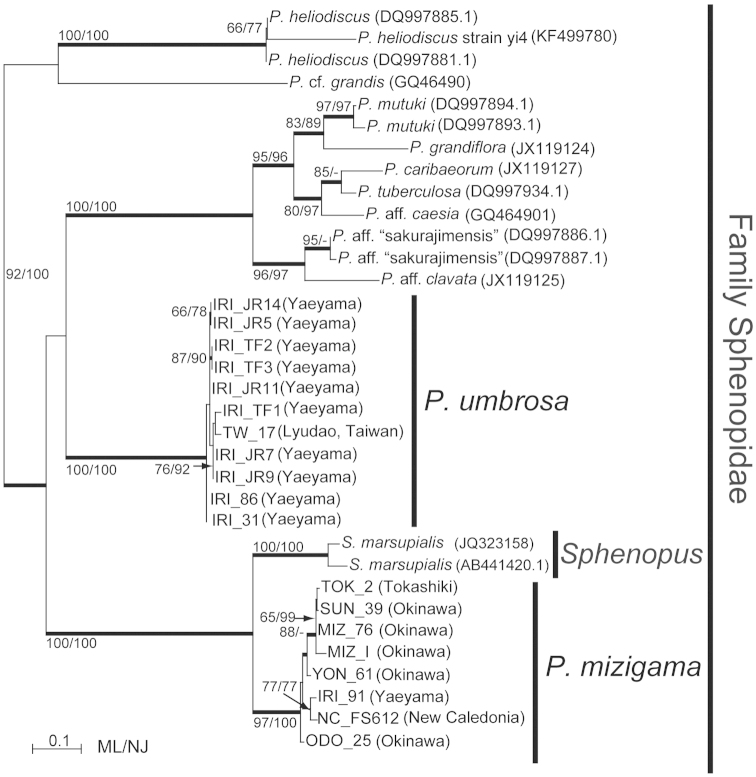
Maximum likelihood tree based on internal transcribed spacer region of ribosomal DNA for family Sphenopidae. Numbers on nodes represent ML and neighbor-joining (NJ) bootstrap values (>65% are shown). Thick branches indicate high supports of Bayesian posterior probabilities (>0.95). Sequences obtained from GenBank are shown with accession numbers.

**Figure 8.**
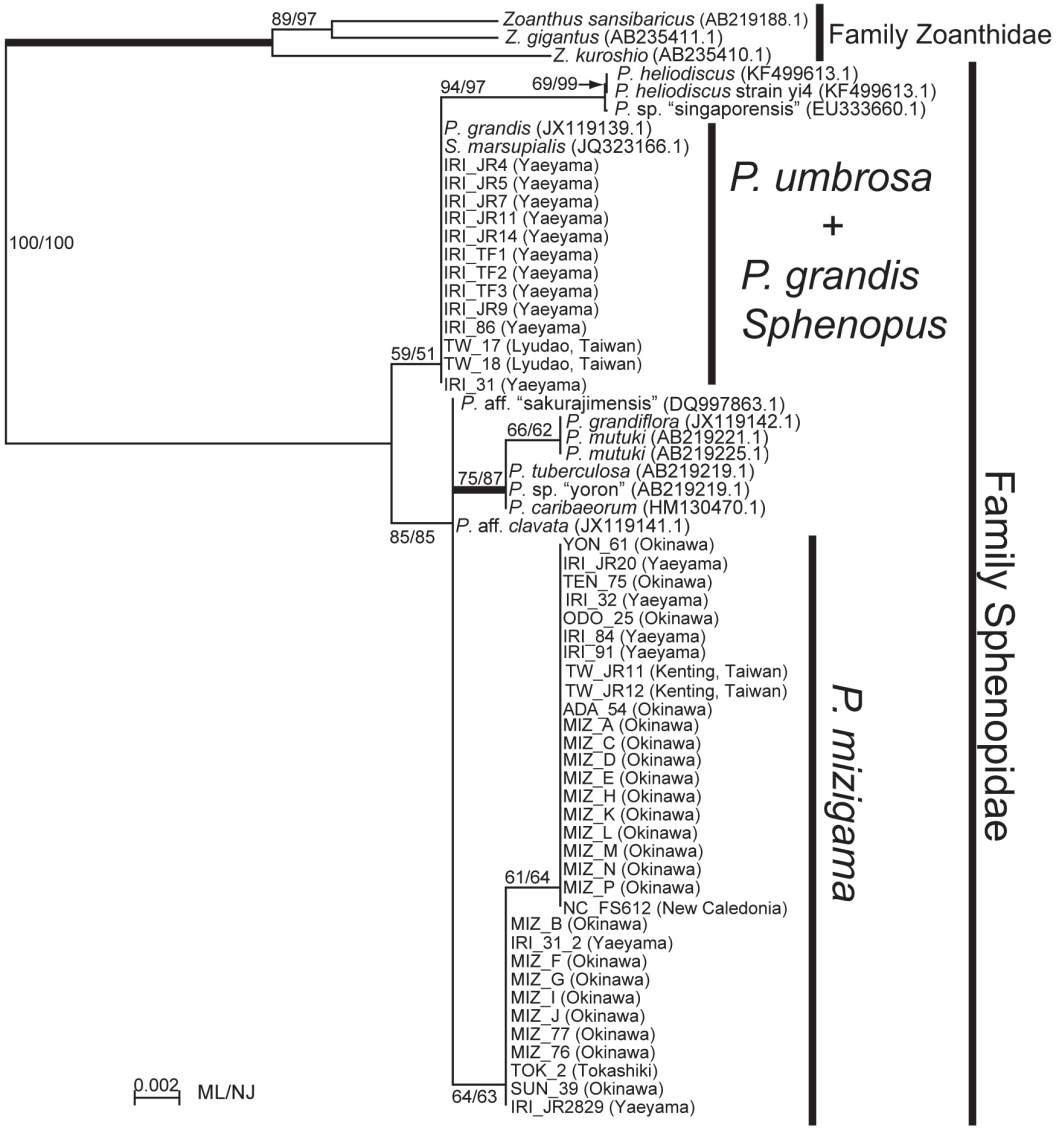
Maximum likelihood tree based on mitochondrial 16S ribosomal DNA for family Sphenopidae. Numbers on nodes represent ML and neighbor-joining (NJ) bootstrap values (>50% are shown). Thick branches indicate high supports of Bayesian posterior probabilities (>0.95). Sequences obtained from GenBank are shown with accession numbers.

**Figure 9.**
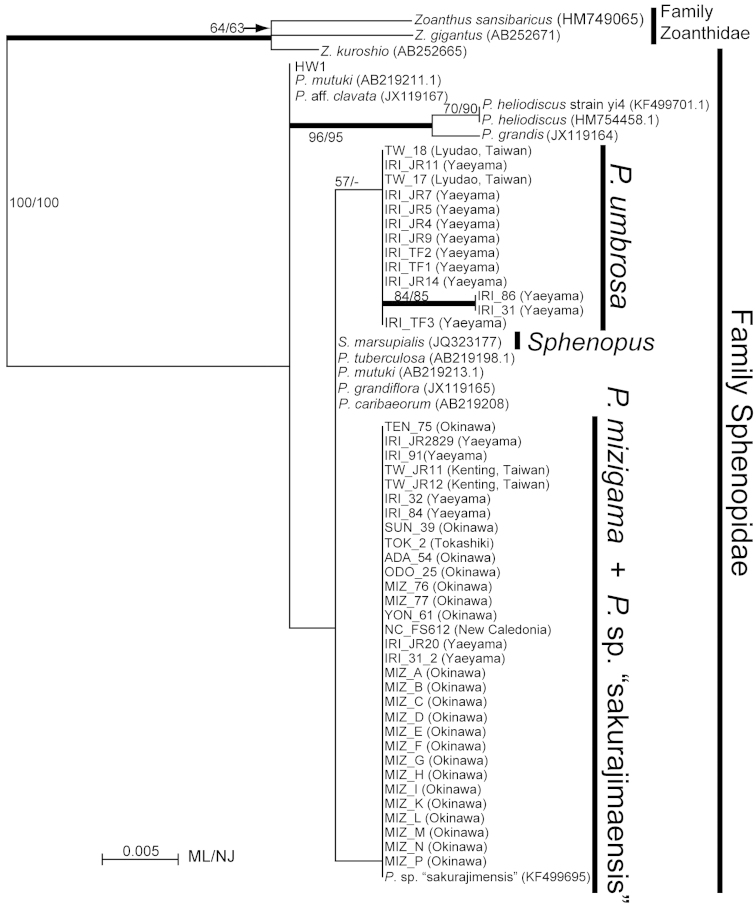
Maximum likelihood tree based on mitochondrial cytochrome oxidase subunit I gene for family Sphenopidae. Numbers on nodes represent ML and neighbor-joining (NJ) bootstrap values (>50% are shown). Thick branches indicate high supports of Bayesian posterior probabilities (>0.95). Sequences obtained from GenBank are shown with accession numbers.

Figures 1 , 2 , 4 , 6 , 7 , 8 , 9 , Suppl. material 1 –2 : Table 1 –2

“Palythoa sp. tokashiki” – Reimer et al. 2011b (specimen from Lyudao (=Green Island), Taiwan), Reimer et al. 2013 (specimen from Lyudao). Synonymy.

##### Type material.

**Type-specimens.** Holotype. Specimen number NSMT-Co1561 (original number IRI_TF3). Split into two pieces and fixed in 99.5% ethanol and 4-10% formalin, respectively. Deposited in National Museum of Nature and Science, Tokyo, Japan. Original label: “HOLOTYPE *Palythoa umbrosa*, Japan, Yaeyama Islands, Sotobanari Island, 8 m depth, 9 November 2012, J. Reimer leg.” Paratypes. Paratype 1. Specimen number RMNH Coel. 41730 (original number IRI_TF1), Japan, Okinawa Prefecture, Yaeyama Islands, Taketomi, Sotobanari Island, 24°22'46"N, 123°43'53"E, at 9 m depth, 9 November 2012, T. Fujii leg. Split into two pieces and fixed in 99.5% ethanol and 4–10% formalin, respectively. Deposited in Naturalis Biodiversity Center, Leiden, The Netherlands. Paratype 2. Specimen number USNM 1231376 (original number IRI_TF2), Japan, Okinawa Prefecture, Yaeyama Islands, Taketomi, Sotobanari Island, 24°22'46"N, 123°43'53"E, at 10 m depth, 9 November 2012, T. Fujii leg. Split into two pieces and fixed in 99.5% ethanol and 4–10% formalin, respectively. Deposited in Smithsonian Institution National Museum of Natural History, Washington, D.C., USA. Paratype 3. Specimen number RUMF-ZG-04376 (original number IRI_31), Japan, Okinawa Prefecture, Yaeyama Islands, Taketomi, Iriomote-suido, 24°21'51"N, 123°57'25"E, at 6 m depth, 7 May 2008, Y. Irei leg. Fixed in 99.5% ethanol, deposited in University Museum, University of the Ryukyus (Fujukan).

##### Type-locality.

Japan, Okinawa Prefecture, Yaeyama Islands, Taketomi, Sotobanari Island, 24°22'46"N, 123°43'53"E, on wall of reef cave at 8 m depth, 9 November 2012, J. Reimer leg.

##### Description of holotype.

Size of colony approximately 2 cm × 2 cm, consisting of three polyps, 6.1 mm in height and 2.7–3.0 mm in diameter. Columns yellowish white with irregular black blotches. Dark brown polyp heads. Horizontal wrinkles (3–5 in number) of approximately half the length of column periphery, on mostly inner side of bent polyps. Tentacles approximately 32 in number, white in color. Columns heavily incrusted with irregularly-sized sand grains.

##### Habitat and behavioral features.

This species inhabits low-light environments such as floors or sides of caves, crevasses or under reef overhangs on coral reef flats and reef slopes. In general, polyps are open at night with extended tentacles, and are closed during the daytime. Polyps tend to bend parallel to the surrounding substrate when closed (Figures 2f, 6b) and become erect in a diagonal direction at an acute angle when open, with oral disks generally facing the opening of the cave or crevasse.

##### Distribution.

Southern Ryukyu Archipelago (Yaeyama Islands) and Taiwan (Lyudao) (Figure 1)

##### Diagnosis.

*General.* Azooxanthellate brachycnemic zoantharian with heavily sand-incrusted ectoderm and mesoglea. Colonies usually composed of several to 20 polyps with each polyp loosely connected to neighbor(s) by a thin stolon (=“liberae”, Pax 1910). Solitary polyps are also commonly seen. Polyps are cylindrical and upper portion of polyps around pharynx is often but not always constricted when closed (Figure 6b, d). Columns occasionally have several horizontal wrinkles (1 to 10 in number) of one quarter to half the length of column periphery, on mostly inner side of bent polyps (Figure 6d).

*Polyp size.* 0.5–0.9 cm in length and 0.3–0.5 cm in width after fixation in 4–10% seawater formalin or 70–99.5% ethanol.

*Coloration of polyp column and oral disk.* Since sampling of *Palythoa umbrosa* was performed only during the daytime, images of expanded polyps in situ were not available and observations were performed under a stereomicroscope. The color of external polyp columns was ivory to tan, occasionally with irregular black/brown blotches (Figures 2f, 6a,b). The color of oral disks was also ivory to tan.

*Tentacles.* 32–38 in number and white to ivory in color (Figure 6a). Occasionally, very small (<0.01 mm) black dots are present (Figure 6c). No stripes on tentacles, unlike *Palythoa mizigama*.

*Cnidom.* There were four major cnidae types observed (Figure 4). The dominant types of cnidae were spirocysts (length 21.9±3.1 μm, width 3.5±0.7 μm, n=80) in the tentacles, basitrichs (length 29.5±3.3 μm, width 3.7±0.4 μm, n=80) in the pharynx, and *p*-mastigophores (length 20.5±3.9 μm, width 5.5±0.9 μm, n=80) and holotrichs (length 13.3±1.5 μm, width 5.0±1.1 μm, n=55) in the mesenterial filaments (Suppl. material 2: Table 2). For all cnidae types except holotrichs, the average lengths were significantly longer in *Palythoa umbrosa* than in *Palythoa mizigama* (t-test *p*<0.05). However, there were relatively large overlaps in the range of lengths between these two species (Suppl. material 2: Table 2).

##### Etymology.

Named for habitat environment of this species. The specific epithet “umbrosa” means “shadowy” in latin. Japanese name: Shirote-yami-iwasunaginchaku. “Shirote” means “white hands” in Japanese.

##### Remarks.

The azooxanthellate species *Palythoa macmurrichi* has previously been described from the Torres Strait, northern Australia. It was described based on a single polyp collected from a channel at 20 fathoms (approximately 36.5 m), and the holotype was lost when it was used for histological examination in Haddon and Shackleton (1891). While the few figures of *Palythoa macmurrichi* in the original description could be either of the two new species in this study, they could also be any of many other *Palythoa* species with “liberae” morphology (Pax 1910). Unfortunately, we are unable to examine *Palythoa macmurrichi* specimens, as this species has not been mentioned in the literature since Verrill (1900) (asides in nomenclators such as Walsh 1967). The current two new species differ from *Palythoa macmurrichi* in their smaller polyp sizes and external markings. Additionally, the two *Palythoa* species in this study were found only in shallow areas (<12 m depth), different from *Palythoa macmurrichi*, which was collected from ~37 m depth. Our SCUBA surveys in Okinawa and New Caledonia investigated channels of similar depths as the type locality of *Palythoa macmurrichi* but we never came across any similar specimens. Thus, for now, we consider *Palythoa macmurrichi* to be a different species from the two new azooxanthellate species described in this study.

### Phylogenetic analyses

*Palythoa mizigama* and *Palythoa umbrosa* were included in a monophyletic clade along with other *Palythoa* spp. in the phylogenetic trees for all three DNA markers (Figures 7–9). Despite the high similarity of the morphological and ecological features of *Palythoa mizigama* and *Palythoa umbrosa*, their sequences consistently formed two separate clades with high support (ML=97%, NJ=100%, Bayes=1.00, and ML=100%, NJ=100%, Bayes=1.00, respectively) in ITS rDNA alignment analyses (Figure 7). The 16S rDNA and COI regions were more conservative than the ITS rDNA region (2–4 bp differences between *Palythoa mizigama* and *Palythoa umbrosa*) and bootstrap scores for each species’ monophyly were not particularly high, but both trees had similar topologies to the ITS rDNA tree, showing the separation of the specimens collected in this study into different clades (Figures 8 and 9, respectively). Previously published sequences of *Sphenopus marsupialis* (Fukami et al. 2008, Reimer et al. 2012b) were also included in the monophyletic clade of genus *Palythoa* for all the three DNA markers (Figures 7–9), and the phylogenetic tree of the ITS rDNA region showed a sister relationship between *Sphenopus* and *Palythoa mizigama* with high bootstrap support (ML=100%, NJ=100%, Bayes=1.00) (Figure 7). On the other hand, the 16S rDNA sequence of *Sphenopus marsupialis* was identical to the sequences of *Palythoa umbrosa* (Figure 8).

## Discussion

### Distribution of *Palythoa mizigama* and *Palythoa umbrosa*

This study revealed a wide distribution for *Palythoa mizigama* in the Central Indo-Pacific from the Ryukyu Archipelago in the north to New Caledonia in the south. *Palythoa umbrosa* was found from a more limited region including the Yaeyama Islands (southwest Ryukyu Archipelago) and Lyudao (= Green Island, southeast of Taiwan) (Figure 1). Even after repeated sampling around Okinawa Island, we did not find a single specimen of *Palythoa umbrosa* from this region. Therefore, it is likely that this species is not present around Okinawa Island. On the other hand, *Palythoa umbrosa* was more common than *Palythoa mizigama* around Yaeyama Islands. Remarkably, at the Iriomote-suido site in the Yaeyama Islands, a *Palythoa mizigama* specimen (specimen IRI_31_2) was found occurring directly next to a *Palythoa umbrosa* colony (specimen IRI_31). Thus, it can be concluded that *Palythoa mizigama* and *Palythoa umbrosa* are sympatric in the Yaeyama Islands, although the overall distributions of *Palythoa mizigama* and *Palythoa umbrosa* are different (Figure 1).

*Palythoa mizigama* and/or *Palythoa umbrosa* may possibly also occur in the Great Barrier Reef, Australia. Burnett et al. (1997) mentioned a *Protopalythoa* (= “liberae” form *Palythoa*) species (*Protopalythoa* sp. 3) with “polyps white or sandy colored, partially buried in coral rock”. Burnett et al. (1997) performed allozyme electrophoretic analyses on zoantharians collected from northwestern Australia and included keys for species identification. The results of UPGMA cluster analyses and descriptions of morphological features in the study lead us to conclude that *Protopalythoa* sp. 3 generally matches well with both *Palythoa mizigama* and *Palythoa umbrosa*. However, it is difficult to confirm that *Protopalythoa* sp. 3 is either *Palythoa mizigama* or *Palythoa umbrosa* as the morphological descriptions in Burnett et al. (1997) were not detailed and the molecular methods used in their study are different from those utilized in our study. Sampling in the current study was mainly focused in the Ryukyu Archipelago, and further investigations in other regions are needed to properly ascertain the overall distributions of these two new *Palythoa* species.

### Distinction between *Palythoa mizigama* and *Palythoa umbrosa*

Both morphological and molecular characteristics clearly indicate the two new species belong within the genus *Palythoa*. Phylogenetic analyses showed that *Palythoa mizigama* and *Palythoa umbrosa* are relatively distantly related to each other within the genus despite their morphological features being almost identical. We detected statistically significant differences in sizes of cnidae between the two species, however, due to large overlaps and relatively high standard deviation values (Suppl. material 2: Table 2), we do not consider cnidae sizes alone as appropriate to distinguish between these species.

The only morphological characteristic that was consistently found to be useful for species identification was tentacle coloration. Specimens of *Palythoa mizigama* and *Palythoa umbrosa* both possessed white tentacles, but black horizontal stripes were always present on the tentacles of *Palythoa mizigama* (Figure 3b–d). On the other hand, *Palythoa umbrosa* specimens had either completely white tentacles or white tentacles with very small black dots that were placed randomly without forming any stripe pattern (Figure 6c). It is recommended to use a stereomicroscope for accurate identification of *Palythoa mizigama* and *Palythoa umbrosa* as *Palythoa mizigama* polyps with sparse stripes on the tentacles (Figure 3d) are hard to distinguish from *Palythoa umbrosa* when observed with the naked eye.

In general, species identification based on coloration has been thought of as not generally reliable for brachycnemic zoantharians as much intraspecific variation is present (Burnett et al. 1995, 1997, Reimer et al. 2004). However, this study suggests that tentacle coloration in *Palythoa mizigama* and *Palythoa umbrosa* reflects their status as separate species, and this character may be useful for rapid field identification of *Palythoa mizigama* and *Palythoa umbrosa*. The utility of coloration has also been mentioned in Sinniger and Häussermann (2009) for macrocnemic zoantharians, which tend to have more uniform coloration. Unlike many zooxanthellate brachycnemic zoantharians, *Palythoa mizigama* and *Palythoa umbrosa* have relatively plain coloration with limited intraspecific variation (e.g. white to tan base color with or without black blotches at random locations on the column). Naturally, polyp columns and oral discs tend to attract more attention than tentacles, as tentacles are generally thin and hard to observe in situ when polyps are closed. Therefore, we recommend that this characteristic not be overlooked, as it is useful for identification in at least these two species.

### Causes of morphological and ecological similarities of the two new *Palythoa* species

This study showed a high morphological similarity between *Palythoa mizigama* and *Palythoa umbrosa* in spite of their relatively distant genetic relationship within genus *Palythoa*. Two possibilities can be considered as the potential cause of this situation. One hypothesis is that *Palythoa mizigama* and *Palythoa umbrosa* have recently diverged from each other but their morphological characteristics have not yet diverged. However, our phylogenetic results showed that *Palythoa mizigama* and *Palythoa umbrosa* do not form a monophyletic group with any of three DNA markers used in this study, and even conservative mitochondrial DNA regions (COI and 16S rDNA) differentiated between the two groups. Thus, there is no support for the idea that *Palythoa mizigama* and *Palythoa umbrosa* have recently diverged.

The other hypothesis is that *Palythoa mizigama* and *Palythoa umbrosa* diverged at relatively the same time as many other zooxanthellate *Palythoa* spp. (e.g. *Palythoa mutuki*, *Palythoa heliodiscus*, *Palythoa tuberculosa* in Figures 7–9), and their unique yet similar morphological and ecological characteristics developed independently in each species as a case of parallel evolution. *Palythoa mizigama* and *Palythoa umbrosa* are azooxanthellate, and this trait is previously almost completely unknown within *Palythoa*. The phylogenetic trees obtained in this study suggested that the two species lost their zooxanthellae separately (Figs 7–9). It is apparent that their habitat in caves and fissures has had a strong influence on the losses of zooxanthellae. Loss of zooxanthellae in relation to cave dwelling anthozoans has also been reported from a recently described cave-dwelling scleractinian species (Hoeksema 2012). Other brachycnemic zoantharians are all zooxanthellate asides from *Sphenopus* spp. and *Palythoa macmurrichi* (Haddon & Shackleton, 1891) and obtain at least part of their nutritional input from photosynthesis, with the remainder from plankton feeding (Trench 1974). *Palythoa mizigama* and *Palythoa umbrosa* inhabit dim environments where enough sunlight for photosynthesis is not available and thus their source of nutrients appears to be exclusively planktonic. Long tentacles have advantages for plankton feeding (Koehl 1977, Sebens 1981), and are seen in other *Palythoa* species such as *Palythoa variabilis* (Duerden, 1898) (Koehl 1977) and in azooxanthellate, planktonivoric macrocnemic species. White, plain coloration might be due to the lack of zooxanthellae or a lack of host-based pigments that protect animals from UV light (Salih et al. 2000), and a similar lack of coloration has been noted from a wide variety of azooxanthellate Zoantharia species (e.g. *Microzoanthus kagerou* Fujii & Reimer, 2011, *Abyssoanthus nankaiensis* Reimer & Fujiwara, 2007, and many *Epizoanthus* species). There are other characteristics that were observed in the two *Palythoa* species such as non-erect polyps when closed, or black patterning on the external polyps, though advantages or causes of these characteristics are unclear. It is possible that some of the characteristics seen in the two species are symplesiomorphies, inherited from a common ancestor. However, there would also be other factors influencing morphologies of the two species, considering that these characteristics are rare among brachycnemic zoantharians. A combination of relatively similar genomic information and a common habitat may have resulted in large morphological overlaps of the two new species in this study.

### Relationship between *Sphenopus* and *Palythoa*

*Sphenopus* has an unusual morphology and ecology among zoantharians, as it is solitary and buried in sand or soft substrate usually without attaching to the substrate. From these unique characteristics, *Sphenopus* has been considered to be a different genus from *Palythoa*. However, the general morphological characters (e.g. mesenterial arrangement, heavy sand incrustation etc.) of *Sphenopus* asides from the lack of colonial form and lack of attachment are the same as for *Palythoa*. In fact, there are some *Palythoa* species that have somewhat similar ecological features to *Sphenopus*, such as *Palythoa psammophilia* Walsh & Bowers, 1971, which inhabits sandy areas with its polyps partially buried (Walsh and Bowers 1971). Other examples are the two new azooxanthellate species in this study with polyps often found in solitary form. Additionally, the results of current and previous (Reimer et al. 2012b) phylogenetic analyses indicate that genus *Sphenopus* is genetically positioned within the genus *Palythoa* (Figures 7–9). Arrigoni et al. 2014 also showed that colony form (solitary or colonial) or possession of zooxanthellae does not always reflect the phylogenetic relationship in the family Dendrophylliidae (Scleractinia). These traits probably change quickly by responding to the surrounding environments. Therefore, considering the morphological and ecological overlaps and the phylogenetic results, it may be appropriate to include *Sphenopus* within *Palythoa*. Surprisingly, *Sphenopus marsupialis* showed a sister relationship with *Palythoa mizigama* in the phylogenetic tree of ITS rDNA region (Figure 7), although validity of this result is still somewhat uncertain as it was not supported in the 16S rDNA and COI trees.

## Conclusions

Planktonivory (Fabricius and Metzner 2004) combined with an association with *Symbiodinium* spp. is a typical ecological feature of species of genus *Palythoa*. From the discovery of the two new azooxanthellate *Palythoa* species in this study, and considering genus *Sphenopus* should likely be included within genus *Palythoa*, it is clear *Palythoa* encompasses a more ecologically diverse group of species than previously understood. From our phylogenetic results it appears that a loss of *Symbiodinium* symbioses can occur relatively quickly on an evolutionary time scale at the level of individual species. These results further demonstrate how ecological traits in Zoantharia previously considered to be important at higher taxonomic levels (genus, family) may evolve and change more rapidly than has generally been assumed. Similar results have been seen in the losses of sand incrustation (Reimer et al. 2011a) and skeleton secretion (Sinniger et al. 2013). Investigations on the relationships between phylogeny and habitats of *Palythoa* spp. and *Sphenopus* spp. might be helpful to track the evolutionary history of family Sphenopidae.

The two new species have very similar morphological features although their phylogenetic positions are relatively distant and without molecular analyses they could have been misdiagnosed as a single species. To avoid taxonomic confusion of zoantharian identification caused by their morphological plasticity, a combination of molecular and morphological data is recommended (Swain and Swain 2014). As seen in previous studies (Sinniger et al. 2008, Reimer et al. 2012a), the combination of the three DNA markers (mitochondrial COI and 16S rDNA, ITS rDNA) utilized in this study was sufficient to distinguish between species. In this study, conservative mt DNA (Huang et al. 2008) differentiated between the new species, even though a few species share identical sequences with the two new species for some of these regions (e.g. COI sequences of *Palythoa mizigama* and Palythoa sp. sakurajimensis are identical, and 16S rDNA sequences of *Palythoa umbrosa*, *Palythoa grandis*, and *Sphenopus marsupialis* are identical) (Figures 8 and 9). Although ITS rDNA is useful for species-level identification, development of additional new DNA markers is needed for furthering research and obtaining more robust data, which will help elucidate more detailed intrageneric zoantharian phylogenies.

## Supplementary Material

XML Treatment for Palythoa

XML Treatment for Palythoa mizigama

XML Treatment for Palythoa umbrosa

## Supplementary materials

Supplementary material 1 Table 1 Data type: Microsoft Excel File (xlsx) Explanation note: List of *Palythoa mizigama* sp. n. and *Palythoa umbrosa* sp. n. specimens used in molecular analyses. This dataset is made available under the Open Database License (http://opendatacommons.org/licenses/odbl/1.0/). The Open Database License (ODbL) is a license agreement intended to allow users to freely share, modify, and use this Dataset while maintaining this same freedom for others, provided that the original source and author(s) are credited.Yuka Irei, Frederic Sinniger, James Davis Reimer

Supplementary material 2 Table 2 Data type: Microsoft Excel File (xlsx) Explanation note: Length and widths of four major cnidae types found in *Palythoa mizigama* sp. n. and *Palythoa umbrosa* sp. n. This dataset is made available under the Open Database License (http://opendatacommons.org/licenses/odbl/1.0/). The Open Database License (ODbL) is a license agreement intended to allow users to freely share, modify, and use this Dataset while maintaining this same freedom for others, provided that the original source and author(s) are credited.Yuka Irei, Frederic Sinniger, James Davis Reimer
